# Antimicrobial Activity and Mechanisms of Walnut Green Husk Extract

**DOI:** 10.3390/molecules28247981

**Published:** 2023-12-07

**Authors:** Lei Wang, Wenjing Li, Xuanyue Li, Jiancheng Liu, Yong Chen

**Affiliations:** Xinjiang Key Laboratory of Herbivore Nutrition for Meat & Milk, College of Animal Science, Xinjiang Agricultural University, Urumqi 830052, China; xjauwl@163.com (L.W.); hlz080@163.com (W.L.); xjaulxy@126.com (X.L.); liujc@xjau.edu.cn (J.L.)

**Keywords:** walnut green husk extract, antibacterial activity, transcriptome analysis, RNA degradation, excessive DNA replication, energy metabolism

## Abstract

Walnut green husks (WGHs), by-products of walnut production, are believed to possess antimicrobial properties, making them a potential alternative to antibiotics. In this study, the antibacterial activities of three extracts, derived from WGH, against *Staphylococcus aureus*, *Bacillus subtilis*, and *Escherichia coli* were investigated, and the antibacterial mechanisms of an anhydrous ethanol extract of WGH (WGHa) were examined. The results showed that WGHa exhibited inhibitory effects on all tested bacteria. The ultrahigh-performance liquid chromatography–tandem mass spectrometry analysis revealed that the major active compounds present in WGHa were terpenoids, phenols, and flavonoids. Treatment with WGHa resulted in the leakage of intracellular ions and alkaline phosphatase; a reduction in intracellular ATP content, ATPase activity, and nucleic acid content; as well as cellular metabolic viability. The transmission electron microscopy images showed varying degrees of cell deformation and membrane damage following WGHa treatment. The transcriptome sequencing and differentially expressed gene enrichment analyses revealed an up-regulation in pathways associated with RNA degradation, translation, protein export, and oxidative phosphorylation. Conversely, pathways involved in cell movement and localization, as well as cell wall organization and carbohydrate transport, were found to be down-regulated. These findings suggest that WGHa alters cell membrane permeability and causes damage to the cell wall. Additionally, WGHa interferes with cellular energy metabolism, compromises RNA integrity, and induces DNA replication stress, consequently inhibiting the normal growth and proliferation of bacteria. These findings unveiled the antimicrobial mechanisms of WGHa, highlighting its potential application as an antibiotic alternative.

## 1. Introduction

Antibiotics have played a crucial role in preventing, controlling, and treating infectious diseases caused by bacteria and fungi [1]. However, the emergence of antibiotic resistance has become a global health concern. The effectiveness of the currently available antibiotics is being challenged by the rise in antibiotic-resistant bacteria [2]. To address this issue, researchers are exploring alternative strategies, and one promising avenue is the utilization of plant secondary metabolites as substitutes for antibiotics. Plant extracts containing phytochemicals have shown immense potential in treating various infectious diseases and have been tested against a wide range of bacteria, including common and antibiotic-resistant strains [3]. Additionally, plant extracts are generally considered environmentally friendly and pose minimal risk of adverse effects on humans and animals. Nowadays, plant-based products are being utilized in healthcare, food preservation, animal production, and crop protection in many countries, demonstrating promising prospects for further applications [4,5,6,7].

Walnuts (*Juglans regia* L.) are widely regarded as one of the healthiest foods in the world. They are abundant in unsaturated fatty acids, minerals, antioxidants, and essential vitamins, which contribute to human well-being [8]. According to data from the United States Department of Agriculture (USDA), global walnut production is projected to reach approximately 2.6 million tonnes (in-shell basis) in the 2022/2023 period. China, as the leading producer, is expected to account for 1.4 million tonnes, representing 53.8% of global production in 2022/2023 [9].

Walnut green husks (WGHs) are the predominant by-product derived from walnut processing, representing 45–60% of the weight of fresh walnut fruit [10]. This by-product has long been utilized in traditional medicine for the treatment of dermatological issues, pain, diarrhea, and diabetes. Walnuts are abundant in plant secondary metabolites, and extensive research using GC-MS and HPLC-TMS techniques has identified a multitude of active components in WGHs, as well as in walnut leaves, bark, and *Diaphragma juglandis fructus*. The apolar compounds include alkanes, alkenes, naphthoquinones, terpenes, sterols, and fatty acids, whereas the principal polar compounds consist of polyphenols and flavonoids [10,11]. The ethyl acetate polar fraction (EAPF), derived from WGH extracts, has displayed excellent microbial activity against both *Escherichia coli* and *Bacillus cereus*, as well as significant radical scavenging capabilities toward 2,2-diphenyl-1-picrylhydrazyl (DPPH), hydroxyl radical (·OH), and 2,2-azino-bis-3-ethylbenzothiazoline-6-sulfonate (ABTS) [11]. Furthermore, WGH extracts have been found to possess NO scavenging abilities, along with the ability to inhibit NO production by a mouse macrophage-like cell line (RAW264.7), indicating its potential anti-inflammatory effects [12]. Notably, WGH extracts or pure extracted compounds from WGHs, such as juglone and terpenes, have been found to exhibit favorable antitumor properties against several human cancer cell lines, including HepG2, MCF-7, HCT-116, HeLa, K562, Raji, and THP-1 [10].

Given its remarkable antimicrobial activity, WGH extracts have the potential to serve as an alternative to antibiotics in various industries such as medicine, food, and animal feed. The composition of active ingredients in WGH extracts can vary widely due to factors such as the variety, growing conditions, harvest time, plant parts used, and extraction solvents. These variations significantly impact the antimicrobial capacity of the extracts. Many polar phytochemicals, including flavonoids and polyphenols, exhibit effective antimicrobial and antioxidant properties. The optimal solvent for antioxidant extraction is 50% hydroalcohol [13]. Although ethyl acetate WGH extracts also exhibit favorable antimicrobial and antioxidant properties [11], water and ethanol are more environmentally friendly. Furthermore, previous studies have focused primarily on the extraction process, the isolation and identification of active ingredients, and the assessment of antimicrobial effects in vitro. However, the antibacterial mechanism of WGH extracts remains unclear.

In this study, the antibacterial abilities of three WGH extracts, WGHa, WGHb, and WGHc, obtained using anhydrous ethanol, 50% hydroethanol, and deionized water, respectively, against *Staphylococcus aureus*, *B. subtilis*, and *E. coli* were evaluated. Based on their antimicrobial performance, WGHa was selected for further evaluation of its impact on the morphology, structure, and function of the aforementioned bacteria. To shed light on the antimicrobial mechanisms of the WGHa against *E. coli*, we conducted a transcriptome sequencing analysis. The results of this study can provide insights into the antimicrobial properties of WGH extracts and contribute to the understanding of their potential applications in food preservation, pharmaceuticals, or other areas where antimicrobial agents are required.

## 2. Results and Discussion

### 2.1. Antimicrobial Activity

#### 2.1.1. Inhibition Zones

**Figure 1:**
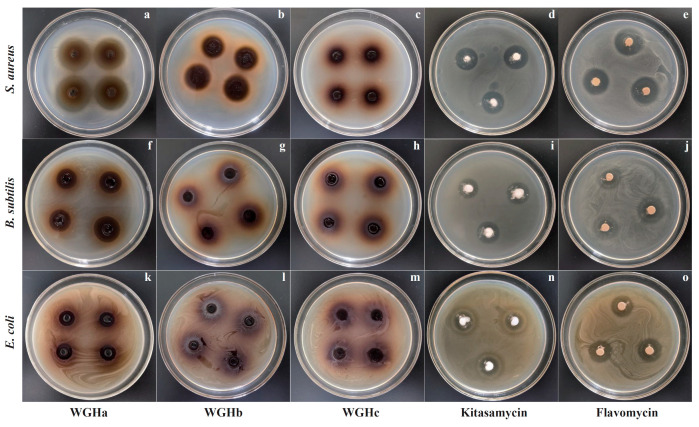
Inhibition of *S. aureus*, *B. subtilis*, and *E. coli* via WGH extracts, kitasamycin, and flavomycint**.** (**a**–**e**) Inhibition zone images of WGHa, WGHb, WGHc, kitasamycin, and flavomycin against *S. aureus*, respectively; (**f**–**j**) inhibition zone images of WGHa, WGHb, WGHc, kitasamycin, and flavomycin against *B. subtilis*, respectively; (**k**–**o**) inhibition zone images of WGHa, WGHb, WGHc, kitasamycin, and flavomycin against *E. coli*, respectively.

The inhibition zones produced by three WGH extracts at a concentration of 200 mg/mL against *S. aureus*, *E. coli*, and *S. aureus* are shown in Table 1 and Figure 1. WGHc did not exhibit any antimicrobial activity against the three selected bacteria. Only *S. aureus* was affected by WGHb, while all three bacteria were susceptible to WGHa. Compared to kitasamycin and flavomycin, WGHa showed greater antimicrobial activity against *S. aureus* and lower antimicrobial activity against *B. subtilis* and *E. coli*. A previous study found that a hexane extract (nonpolar) and hydroethanolic extract (polar) of WGH from seven distinct cultivars exhibited satisfactory antimicrobial activity against both Gram-negative (*E. coli* and *Pseudomonas aeruginosa*) and Gram-positive (*S. aureus*, *B. subtilis*) bacteria [10]. However, *B. cereus*, *B. subtilis*, *S. aureus*, and *S. epidermis* were susceptible to the aqueous extracts of WGHs, whereas *E. coli* and *P. aeruginosa* were not [14]. These discrepancies in antimicrobial activities might be attributed to variations in the extraction methods, applied solvents, plant material, and selected microorganisms [12].

#### 2.1.2. Minimum Inhibitory Concentration (MIC)

The MICs of the three WGH extracts are displayed in Table 2. WGHa exhibited MICs of 6.25 mg/mL against *S. aureus* and *B. subtilis* and 25.00 mg/mL against *E. coli*. The antibacterial activity of WGHb was significantly lower than that of WGHa, with an MIC of 200 mg/mL against *S*. *aureus*. No antimicrobial activity was observed for WGHb and WGHc at concentrations of 100 mg/mL or lower. A previous study found that the MIC for the ethyl acetate fraction of a WGH extract against both *E. coli* and *B. cereus* was 31.25 mg/mL [11]. However, a combination of an n-hexane extract and a 80% hydroethanol extract of WGH exhibited MIC values of 2, 1, 0.5, and 4 mg/mL against *S. aureus*, *B. subtilis*, *E. coli*, and *P. aeruginosa*, respectively [10]. The results of the present study clearly demonstrate that WGHa exhibits significantly better antimicrobial activity than WGHb and WGHc. Taking into consideration these findings, WGHa was chosen for further investigation.

It was found that the incorporation of ZnO nanoparticles significantly reduced the MIC of the WGH extract against *S. mutans* from 50 mg/mL to 3.12 mg/mL [15], indicating that the utilization of nanomaterials can effectively enhance the antimicrobial efficacy of plant extracts. In recent years, hyper-crosslinked polymers (HCPs) have emerged as a promising category of antimicrobial materials due to their customizable structural design and easy functionalization [16]. The L-borneol-loaded HCP nanoparticles exhibited favorable antimicrobial effects against both *E. coli* and *S. aureus* [17]. The incorporation of HCP nanoparticles into WGH extracts may act synergistically to amplify their antimicrobial properties. Given these findings, further attention and investigation are warranted to explore the potential of this novel combination in future research.

#### 2.1.3. Growth Curve

**Figure 2:**
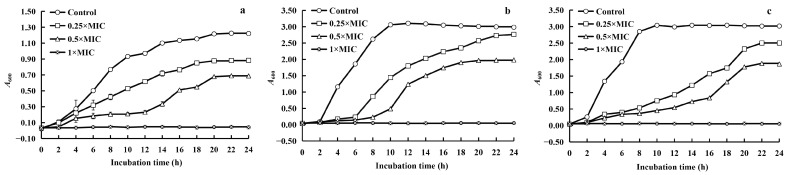
The effect of WGHa on the growth curves of *S. aureus* (**a**), *B. subtilis* (**b**), and *E. coli* (**c**).

The impacts of WGHa on the growth curves of *S. aureus*, *B. subtilis*, and *E. coli* are illustrated in Figure 2.

The addition of WGHa significantly altered the growth rates of the three bacterial strains. Compared to the control, the supplementation of WGHa at doses of 0.25 × MIC and 0.5 × MIC resulted in a noticeable inhibition of the growth curves for all three bacteria, with delayed logarithmic and stable growth phases. Moreover, when WGHa was added at a concentration of 1 × MIC, a near-complete inhibition of all three bacteria was observed. Previous research demonstrated that irrigating *Zea mays*, *Lactuca sativa* cv. Gentilina, and *L. sativa* cv. with walnut washing water can trigger programmed cell death in these crops [18]. Our study results suggest that a low concentration of WGHa hindered the normal proliferation of the selected bacteria during the early stages of incubation, whereas a high concentration caused cell death, indicating a dose-dependent effect of the inhibitory properties of the extract.

### 2.2. Chemical Compositions of WGHa

The chemical composition of WGHa was analyzed using ultrahigh-performance liquid chromatography–tandem mass spectrometry (UHPLC-MS/MS) in both positive and negative ion modes. The total ion chromatograms are shown in Figure S1. A total of 124 compounds were matched by tandem mass spectrometry, with 115 in the positive ion mode and 9 in the negative ion mode. Detailed information on the top 20 compounds in terms of peak area are listed in Table 3.

Among these 20 compounds, 7 were terpenoids, 5 were phenols, and 4 were flavonoids. Previous research employed HPLC-DAD-ESI/MS^n^ to analyze the compounds present in a hydroethanolic extract of WGH. A total of 16 compounds, primarily consisting of naphthalene derivatives such as dihydroxytetralone and trihydroxynaphthalene galloyl-hexoside, were identified. Phenolic compounds were found to be less abundant [12]. In another study, 46 nonpolar compounds were identified in the hexane extract of WGH, including γ-sitosterol, vitamin E, β-tocopherol, lupeol, juglone, oleic acid, linoleic acid, palmitic acid, and stearic acid. Additionally, the 80% hydroethanol extract contained a dozen polar compounds, which included myricitrin, derivatives of quercetin and taxifolin, catechin, abscisic acid, salicylate glucuronide, neochlorogenic acid, and gallic acid [10]. In our current study, a wide array of compounds was identified, with quercetin being the most abundant compound in the positive ion mode, comprising 12.05% of the identified compounds. In the negative ion mode, gallic acid was the most prevalent, accounting for 65.89% of the identified compounds. Furthermore, several bioactive substances, such as chlorogenic acid, cinnamaldehyde, caffeic acid, vanillic acid, epicatechin, ellagic acid, and vanillic acid, were also identified. Importantly, many of these compounds possess antibacterial and antioxidant properties.

### 2.3. Antimicrobial Mechanisms of WGHa

#### 2.3.1. Extracellular Alkaline Phosphatase (AKP) Activity

**Figure 3:**
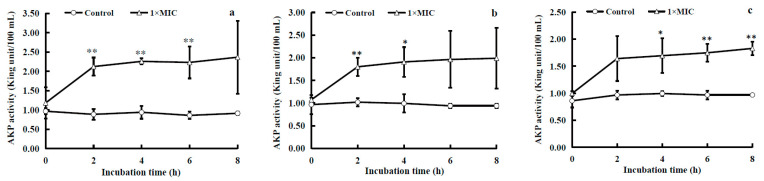
The effect of WGHa on the extracellular AKP activity of *S. aureus* (**a**), *B. subtilis* (**b**), and *E. coli* (**c**). * *p* < 0.05, ** *p* < 0.01.

AKPs (enzyme commission (EC) number: 3.1.3.1) are homodimeric proteins that are initially secreted as monomers into the periplasmic space and situated between the cell wall and cell membrane, where they are dimerized and activated [19,20]. These proteins catalyze the dephosphorylation of nucleic acids, proteins, and alkaloids and play an important role in the cellular uptake of phosphate. Once the cell wall is damaged, the activity of AKPs in the extracellular environment will increase. The effect of WGHa on extracellular AKP activity is shown in Figure 3.

While the blank medium (containing the same amount of WGHa without bacterial inoculation) exhibited consistent AKP activity throughout the incubation period, the experimental groups displayed a sharp increase in extracellular AKP activity within 0 to 2 h of exposure to WGHa. Subsequently, this activity stabilized at a notably high level between 2 and 8 h. AKP catalyzes the hydrolysis of phospholipids to release phosphate [21]. Increased extracellular AKP activity disrupts the cell membrane structure, increasing membrane permeability and leading to more intracellular AKP entering the culture medium. Previous studies have demonstrated that quercetin, the primary active component in WGHa, effectively disrupts the permeability of the bacterial cell membranes of *E. coli* and *S. aureus*, resulting in the leakage of intracellular proteins into the culture medium and elevating the concentration of soluble proteins within the medium [22]. This evidence indicates that the treatment of bacteria with WGHa not only caused damage to the cell wall, loss of cytoderm integrity, and leakage of AKP but also potentially led to the entry of other cellular proteins into the culture medium, consequently impairing cellular functions [11].

#### 2.3.2. Electric Conductivity of the Medium

**Figure 4:**
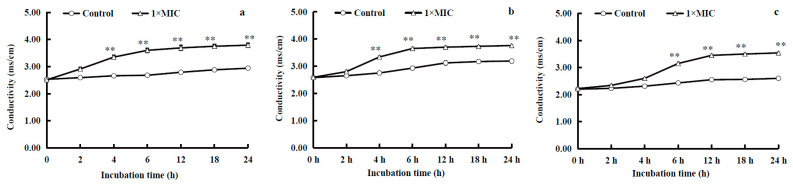
The effect of WGHa on conductivity of the diluted culture media of *S. aureus* (**a**), *B. subtilis* (**b**), and *E. coli* (**c**). ** *p* < 0.01.

As shown in Figure 4, the electric conductivity of the diluted culture solutions of *S. aureus*, *B. subtilis*, and *E. coli* exhibited an increase with increasing incubation time after exposure to WGHa. The electric conductivity of the diluted culture medium of the three tested bacteria in the experimental groups was significantly higher than that of the control groups after incubation for 4 or 6 to 24 h (*p* < 0.01). A previous study discovered that the treatment of *Shewanella putrefaciens* with oregano essential oil resulted in an increase in the conductivity of the culture medium [20]. The observed increase in conductivity was attributed to the efflux of intracellular ions such as Na^+^ and K^+^ as it has been demonstrated that K^+^ efflux values of *B. cereus* and *E. coli* treated with the ethyl acetate polar fraction of a WGH extract were significantly higher compared to that of untreated cells [11,23]. The findings of the present study may be attributed to the interaction between active ingredients such as flavonoids and polyphenols in WGHa and the cell membrane, ultimately leading to an increased cell membrane permeability and the efflux of intracellular substances from the bacterial cell [24,25].

#### 2.3.3. Intracellular ATP Content and ATPase Activity

**Figure 5:**
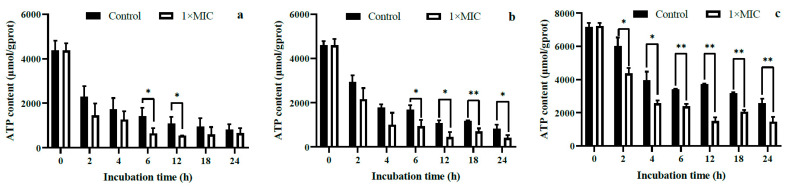
The effect of WGHa on the intracellular ATP content of *S. aureus* (**a**), *B. subtilis* (**b**), and *E. coli* (**c**). * *p* < 0.05, ** *p* < 0.01.

As shown in Figure 5, the intracellular ATP levels of the investigated bacteria exhibited a decline as the duration of incubation increased. Notably, the experimental groups displayed a significantly higher reduction rate compared to the control groups. For *S. aureus*, a significant decrease in intracellular ATP content was observed at 6 and 12 h post-WGHa treatment (Figure 5a), while for *B. subtilis*, this decline extended from 6 to 24 h (Figure 5b), and for *E. coli*, it spanned from 2 to 24 h (Figure 5c). Since ATP is necessary for bacterial cellular processes such as energy conversion, cell proliferation, nutrient metabolism, and the synthesis and transport of biomolecules [26], the reduction in intracellular ATP content implies an inhibition of cell cycle progression. It was found that *Enterobacter hormaechei* cells experienced a significant decrease in intracellular ATP levels upon treatment with vanillic acid [27]. Similarly, caffeic acid, a polyphenol found in WGHa, disrupted ATP synthesis by down-regulating the activity of H^+^-ATPase, consequently impeding the growth of *S. aureus* and *K. pneumoniae* [28]. Additionally, ginger phenolics reduced intracellular ATP synthesis in *E. coli* by inhibiting the activity of F1Fo ATP synthase [29]. Our results suggest that the reduction in intracellular ATP observed in bacteria treated with WGHa may be attributed to ATP leakage resulting from alterations in cell membrane permeability.

**Figure 6:**
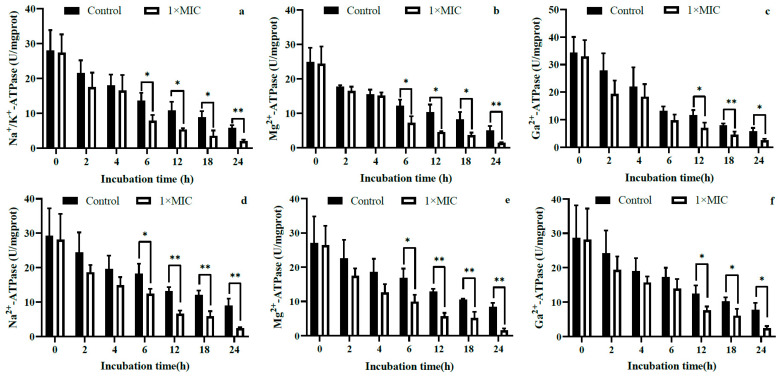

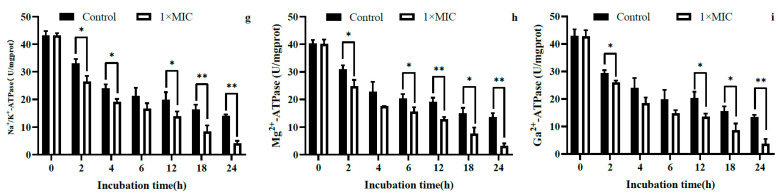
The effect of WGHa on the activity of intracellular ATPases of *S. aureus* (**a**–**c**), *B. subtilis* (**d**–**f**), and *E. coli* (**g**–**i**). * *p* < 0.05, ** *p* < 0.01.

The effects of WGHa on the activity of intracellular Na^+^/K^+^-ATPase, Mg^2+^-ATPase, and Ca^2+^-ATPase are shown in Figure 6. The activities of all three intracellular ATPases in the selected bacteria were inhibited by WGHa and decreased as the incubation time increased. The activities of Na^+^/K^+^-ATPase, Mg^2+^-ATPase, and Ca^2+^-ATPase in *S. aureus* (Figure 6a–c) and *B. subtilis* (Figure 6d–f) were significantly reduced after 6, 6, and 12 h of incubation, respectively, and the differences persisted until the end of the experiment. In *E. coli*, the activity of Na^+^/K^+^-ATPase was significantly decreased at 2, 4, and 12 h after incubation and thereafter (Figure 6g). Similarly, the activity of Mg^2+^-ATPase was significantly decreased at 2 and 6 h after incubation and thereafter (Figure 6h), while the activity of Ca^2+^-ATPase was significantly reduced at 2 and 12 h after incubation and thereafter (Figure 6i). It is worth noting that Na^+^/K^+^-ATPase, Ca^2+^-ATPase, and Mg^2+^-ATPase are important ion channels located on the cell membrane [30]. They play critical roles in intercellular signaling, maintaining cellular homeostasis, regulating the ion concentration gradient between the intra- and extracellular spaces, facilitating normal bacterial metabolism, and preserving the physiological state and adaptability of bacteria. Our findings suggest that compounds in WGHa down-regulate the activity of the ATPases, consequently disrupting cellular ion homeostasis.

#### 2.3.4. Loss of 260 nm Light-Absorbing Substances

**Figure 7:**
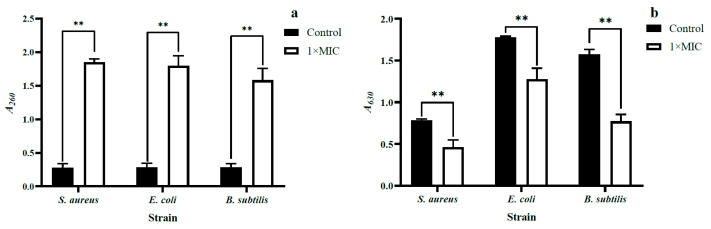
The effect of WGHa on the loss of 260 nm light-absorbing substances (**a**) and cell metabolic activity (**b**) of *S. aureus*, *B. subtilis*, and *E. coli*. ** *p* < 0.01.

When the structure of the bacterial cell membrane is compromised, nucleic acids and other macromolecules within the cell are released, resulting in an increase in absorbance at 260 nm (A260) of the bacterial surrounding environment. Therefore, the A260 can serve as an indicator to evaluate the extent of bacterial lysis [31]. As demonstrated in Figure 7a, following a 20 h treatment with WGHa, the A260 of cell suspensions for *S. aureus*, *B. subtilis*, and *E. coli* were significantly higher compared to the control groups (*p* < 0.01). This observation suggests that certain 260 nm light-absorbing substances (predominantly nucleic acids) leaked out of the cells following the WGHa treatment. A previous study found that quercetin notably facilitated the release of 260 nm light-absorbing substances (mostly DNA, RNA, and metabolites) from the intracellular to the extracellular environment, in comparison to amoxicillin [32]. One of the antimicrobial mechanisms of flavonoids was attributed to their ability to disrupt cell membranes and permeability barriers, consequently causing the leakage of intracellular protein, lipid, and 260 nm light-absorbing substances [33].

#### 2.3.5. Cellular Metabolic Vitality

Iodonitrotetrazolium chloride (INT) can undergo a reaction with H^+^ that is generated by the electron transport system of living cells. This reaction results in the reduction of INT and the production of a stable purple-red formazan. Formazan exhibits a maximum absorbance peak at 630 nm, and the A630 value corresponds positively to the metabolic activity of living cells. Therefore, the A630 value of formazan serves as an indicator to determine the metabolic activity of living cells [34]. As shown in Figure 7b, the A630 of *S. aureus*, *B. subtilis*, and *E. coli* in experimental groups exhibited significantly lower values compared to the respective control groups (*p* < 0.01), indicating a significant reduction in the metabolic viability of the bacteria following treatment with WGHa. The flavonoids and polyphenols present in WGHa have the ability to interact with cell membranes and to penetrate into the cell, causing damage to intracellular components, leading to a decrease in cell viability and, in severe cases, disintegration and death of the bacteria from the inside out [35].

#### 2.3.6. Fluorescence Intensity of Bacterial Nucleic Acids

**Figure 8:**
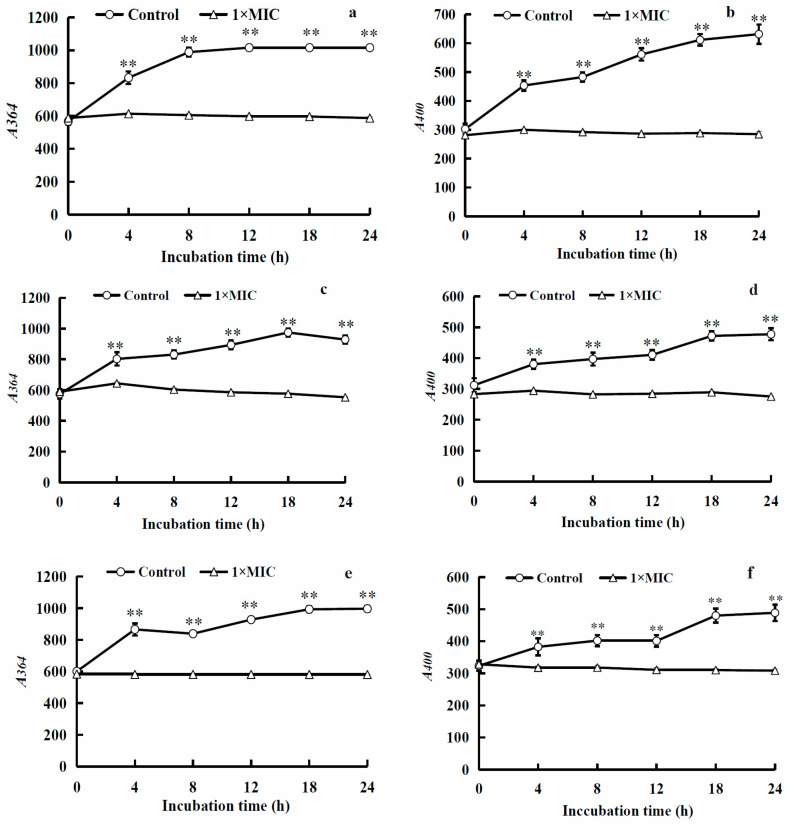
Effects of WGHa on the fluorescence intensity of DNA and RNA of *S. aureus* (**a**,**b**), *B. subtilis* (**c**,**d**), and *E. coli* (**e**,**f**). (**a**,**c**,**e**) DNA fluorescence intensity; (**b**,**d**,**f**) RNA fluorescence intensity. ** *p* < 0.01.

4′,6-diamidino-2-phenylindole (DAPI), a fluorescent dye with the ability to penetrate cell membranes, binds specifically to the minor groove of double-stranded DNA at AT-rich sequences, resulting in the formation of a stable fluorescent complex known as DAPI–DNA. This complex exhibits a fluorescence intensity approximately 20 times greater than that of free DAPI [36]. Although DAPI can also bind to RNA, its fluorescence intensity when embedded in AU sequences of RNA is only around 20% that of the DAPI–DNA complex [37]. The fluorescence intensity of the DAPI–nucleic acid complex is positively associated with the nucleic acid level, making it possible to qualitatively assess the amount of nucleic acid by analyzing the fluorescence intensity of DAPI–nucleic acid complexes in cells. The effects of WGHa on the fluorescence intensity of DNA and RNA of *S. aureus*, *B. subtilis*, and *E. coli* are presented in Figure 8.

Following a 4 h treatment with WGHa, the fluorescence intensity of DAPI–nucleic acid complexes decreased significantly in cells and remained at a low level throughout the incubation period. In contrast, the fluorescence intensity in the control groups continuously increased with increasing incubation time. In general, a high concentration of polyphenols will induce DNA damage; however, a low concentration tends to decrease DNA damage [38]. Phenols extracted from WGH demonstrated notable DNA nuclease activity on pBR322 DNA, with concentrated extracts completely degrading DNA [39]. These observations suggest that WGHa inhibits the synthesis of bacterial nucleic acids or damages bacteria DNA, thereby suppressing bacterial proliferation.

#### 2.3.7. Scanning Electron Microscopy (SEM)

**Figure 9:**
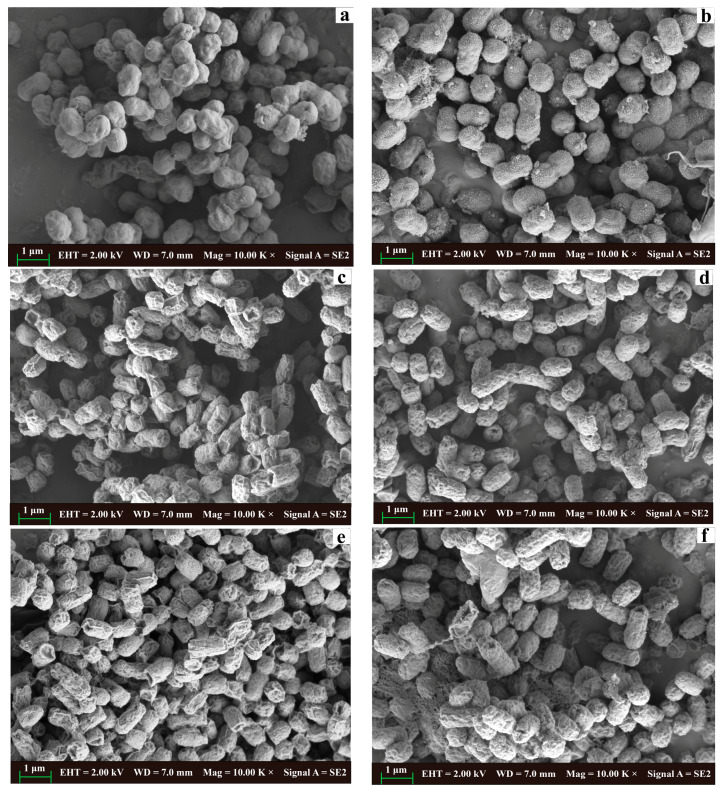
Effect of WGHa on cellular morphology of *S. aureus* (**a**,**b**), *B. subtilis* (**c**,**d**), and *E. coli* (**e**,**f**). (**a**,**c**,**e**) Control groups; (**b**,**d**,**f**) experimental groups. Bacteria in the experimental groups treated with 1 × MIC of WGHa for 20 h. Images were obtained at an accelerating voltage of 2.00 kV and a magnification of 10,000×.

In Figure 9, the treated cells exhibited rougher surfaces alongside various structural alterations, including pores, depressions, and adhesions. Additionally, variations in bacterial size were observed, with some demonstrating dissolution. Comparable findings have been previously reported in antibacterial assessments involving other WGH extracts [11]. Vanillic acid, chlorogenic acid, and cinnamaldehyde, which were found in WGHa, are responsible for these morphological and structural alterations in the cell membranes of the selected bacteria. All these observations point to these compounds causing cell deformation and severe membrane disruption [27,40,41].

### 2.4. Transcriptome Analysis of Differentially Expressed Genes (DEGs)

The results showed that the treatment of *E. coli* with WGHa for 6 h led to a significant reduction in RNA concentration, RNA integrity number (RIN), and the ratio of 23S/16S rRNA compared to the control group (Table S1). The RIN value is commonly used as an indicator to evaluate the integrity of RNA. Higher RIN values indicate better RNA integrity, while lower values suggest more RNA degradation [42]. This decrease in RIN values is consistent with a previous study that demonstrated RNA damage and decreased RIN values in *E. coli* treated with ultra-small gold nanoclusters, a type of nanoantibiotic, for more than 20 min [43]. These findings suggest that WGHa promotes the degradation of RNA to some extent.

After Novaseq 6000 sequencing, a total of 232 million raw reads were obtained from 16 samples. After quality control checks, 226 million clean reads with an average of 14.1 million clean reads per sample were obtained. The clean reads with clean base >2.1 G/sample, Q20 > 98.5%, Q30 > 95%, and a GC content of 52.54% (Table S2) were aligned and mapped to the reference genome of *E. coli* ATCC 8739 using Bowtie2 (v2.5.1). The results showed that, on average, 97.48% of the reads were successfully mapped to the reference genome (ranged from 96.29% to 97.91%) (Table S3).

#### 2.4.1. Identification of DEGs

**Figure 10:**
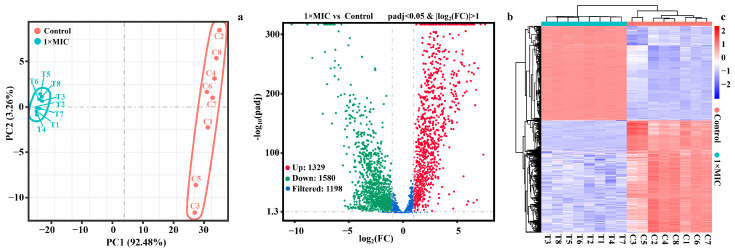
Principal component analysis (PCA) (**a**), volcano diagram (**b**), and heatmap (**c**) of differentially expressed genes (DEGs). (**a**) PCA of gene expression level of *E. coli* treated with WGHa. The *x*-axis represents the first principal component (PC1) and the *y*-axis shows the second principal component (PC2). The distance between data points reflects their similarity, with closer points indicating higher similarity. (**b**) The volcano diagram of DEGs after WGHa treatment. The criteria of significant differential expression were *p*adj < 0.05 and |log_2_(fold change)| > 1. Red plots represent up-regulated genes, green plots represent down-regulated genes, and blue plots represent filtered genes. (**c**) Heatmap of all DEGs. The *x*-axis represents the samples from the control and WGHa-treated groups, and the *y*-axis represents different genes; red means genes exhibiting increased expression, and blue means genes exhibiting decreased expression.

The principal component analysis (PCA), a volcano diagram, and a heatmap of DEGs between the groups are illustrated in Figure 10. The results of the PCA revealed that the gene expression levels of biological replicates were closer within the same group, whereas the gene expression levels of different treatment groups differed significantly and clustered into distinct groups. PC1 and PC2 explained 92.48% and 3.26% of the variance observed in the complete data set, respectively (Figure 10a).

This indicated that WGHa had a significant impact on the gene expression of *E. coli*. Based on the criteria of *p*adj < 0.05 and |log_2_(fold change)| > 1, a total of 2907 DEGs were identified between the control and WGHa-treated groups. Out of these, 1327 genes were up-regulated, while 1580 genes were down-regulated (Figure 10b). The top ten up-regulated genes included four tRNA genes, four ribosomal protein-coding genes, and two genes encoding the pyruvate dehydrogenase E1 component and a stress-induced protein, respectively. On the other hand, the top 10 down-regulated genes primarily included regulators of biofilm formation, stress-induced proteins, and enzymes related to energy metabolism. Additionally, as shown in Figure 10c, the DEGs of samples with different treatments were inconformity. The eight replicates of the WGHa-treated group clustered together, while the control group formed a separate cluster. A complete description of the DEGs is shown in Table S4.

#### 2.4.2. Gene Ontology (GO) Enrichment and Kyoto Encyclopedia of Genes and Genomes (KEGG) Pathway Analysis of DEGs

**Figure 11:**
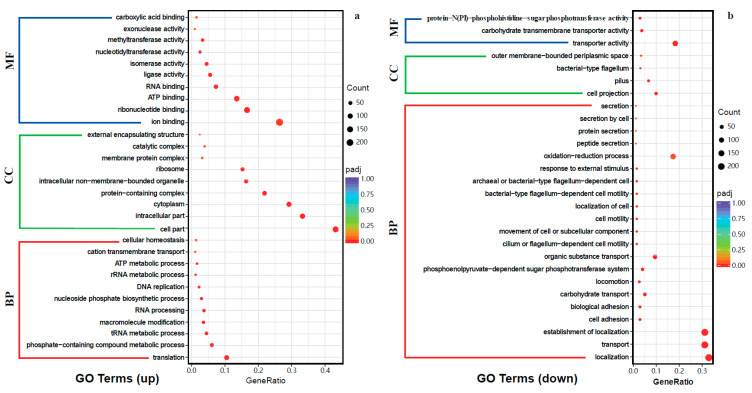

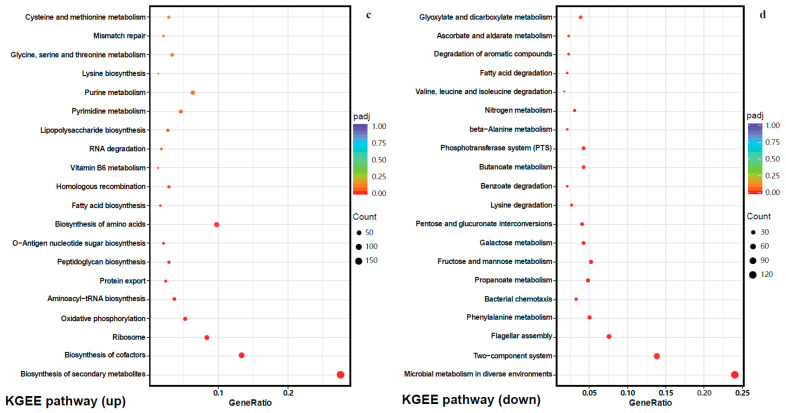
Scatterplot of differential expressed genes by Gene Ontology (GO) enrichment analysis. (**a**) The top 30 up-regulated GO terms; (**b**) the top 30 down-regulated GO terms. DEGs enriched in biological processes (BPs), cellular components (CCs), and molecular functions (MFs); (**c**) the top 20 up-regulated Kyoto Encyclopedia of Genes and Genomes (KEGG) pathways; (**d**) the top 20 down-regulated KEGG pathways. The dots size represents the count of enriched genes.

In the present study, a total of 435 GO terms were enriched, encompassing 254 terms in biological processes (BP), 30 terms in cellular components (CC), and 151 terms in molecular functions (MF). Among the BP terms, translation (GO:0006412), tRNA metabolic process (GO:0006399), and phosphate-containing compound metabolic process (GO:0006796) were significantly up-regulated, while localization (GO:0051179), transport (GO:0006810), and cell adhesion (GO:0007155) were significantly down-regulated. Regarding the CC terms, the up-regulated terms were mainly enriched in the intracellular part (GO:0044424), cell part (GO:0044464), and cytoplasm (GO:0005737), whereas the down-regulated terms were mainly associated with cell projection (GO:0042995) and the pilus (GO:0009289). Concerning the MF terms, 42 terms showed significant up-regulation, with ion binding (GO:0043167), nucleotide binding (GO:0000166), and ATP binding (GO:0005524) being the predominant terms. Only three MF terms exhibited significant down-regulation, which are primarily related to transporter activity (GO:0005215) (Figure 11a,b). These findings shed light on the alterations to numerous genes involved in RNA biosynthesis and metabolism, protein translation and translocation, cell homeostasis and motility, as well as secretion and transmembrane transport of substances. Consequently, these alterations contribute to structural and functional modifications within the cells.

The KEGG enrichment analysis showed that a total of 14 pathways were up-regulated, while 20 pathways were down-regulated in *E. coli* after WGHa treatment. Among these pathways, the biosynthesis of secondary metabolites (ecl01110), biosynthesis of cofactors (ecl01240), and ribosome (ecl03010) were the top three up-regulated pathways, while microbial metabolism in diverse environments (ecl01120), the two-component system (ecl02020), and flagellar assembly (ecl02040) were the top three down-regulated pathways (Figure 11c,d). These findings provide new insight into the potential mechanisms underlying the antibacterial properties of WGHa against *E. coli*.

#### 2.4.3. Gene Set Analysis of DEGs

##### DEGs Related to Cell Structures

**Figure 12:**
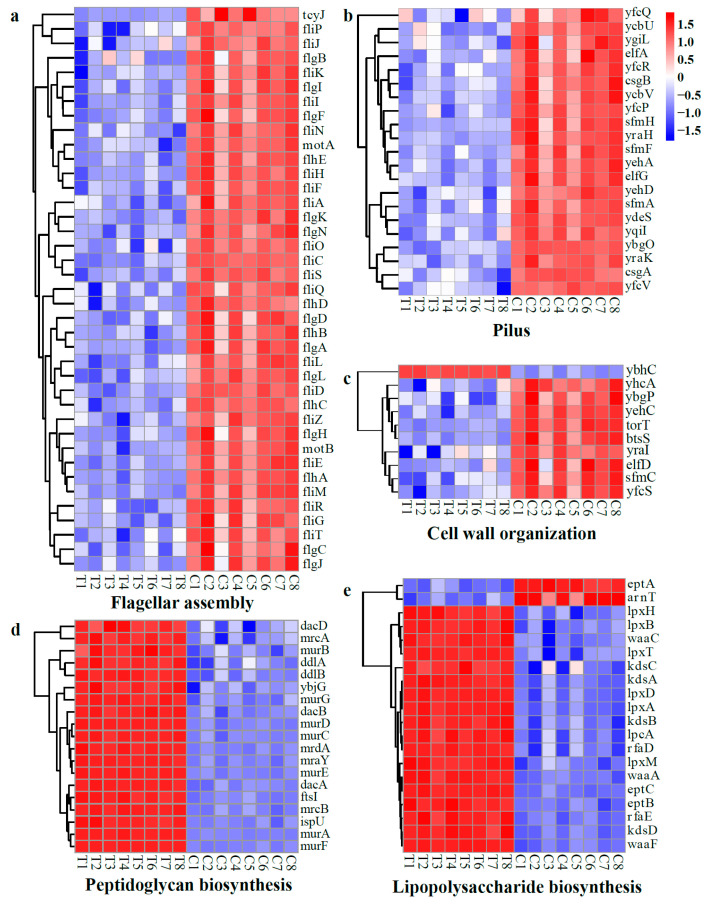
Heat map of typical DEGs related to cell structures, including flagellar assembly (**a**), pilus (**b**), cell wall organization (**c**), peptidoglycan biosynthesis (**d**), and lipopolysaccharide biosynthesis (**e**). Red indicates up-regulated genes, while blue indicates down-regulated genes.

The flagellum, cell wall, and cell membrane are foundational structures of bacteria that serve as crucial protective barriers in cell motility, environmental sensing, and the maintenance of cellular integrity. A gene set analysis of the DEGs revealed that numerous genes responsible for flagellar assembly, the pilus, and cell wall organization of *E. coli* were down-regulated, while multiple genes for the biosynthesis of lipopolysaccharide (LPS) and peptidoglycan (PG) were up-regulated after treatment with WGHa (Figure 12). Notably, the genes coding for flagellar structural protein (*flgB* and *fliC*), flagellar biosynthesis protein (*fliH)*, flagellar transcriptional activator (*flhD*), flagellar motor switch protein (*fliG*) (Figure 12a), fimbrial protein (*elfA*/*G*, *sfmF*/*H*, *ybgO*, *ycbU*/*V*, *ydeS*, *yehA*/*D*, *yfcP*/*Q*/*R*/*V*, *ygiL*/*I*, and *yraH*/*K*) ((Figure 12b), and pili and flagellar-assembly chaperone (*elfD*, *sfmC*, *yehC*, *ybgP*, *yfcS*, *yhcA*, and *yraI*) (Figure 12c) were significantly down-regulated in response to WGHa treatment. The down-regulation of genes encoding fimbriae, pili, and flagellar proteins suggests impaired bacterial adherence, connectivity, motility, and locomotion [44,45]. However, genes encoding carboxypeptidase (*dacA*/*B*/*D*), dipeptidases (*ddlA*/*B*), peptidoglycan glycosyltransferase (*mrcA*/*B*), and N-acetylglucosamine and stem pentapeptide synthase (*murA*/*B*/*C*/*D*/*E*/*F*/*G*), which are involved in PG biosynthesis (Figure 12d), as well as the sulfatase (*eptB*/*C*), acid sugar synthesis and transfer (*kdsA*/*B*/*C*/*D* and *waaA*), acyl transfer (*lpxA*/*B*/*D*/*M*), and LPS synthesis and transport (*lptG*, *rfaD*, and *waaC*) genes in LPS biosynthesis were significantly up-regulated (Figure 12e). LPS plays a fundamental role in bacterial defenses against environmental stressors, drug resistance, pathogenesis, and symbiosis [46]. Furthermore, PG is a major component of the bacterial cell wall, playing a critical role in determining cell shape and enabling bacteria to survive in hypotonic environments [47]. Thus, in response to the stressful effects of WGHa, in a compensatory fashion, *E. coli* up-regulated the genes related to LPS and PG biosynthesis. Therefore, the up-regulation of PG and LPS biosynthesis-related genes reflects the disruptive effects of WGHa on *E. coli* cell walls.

##### DEGs Related to Cell Membrane Functions

**Figure 13:**
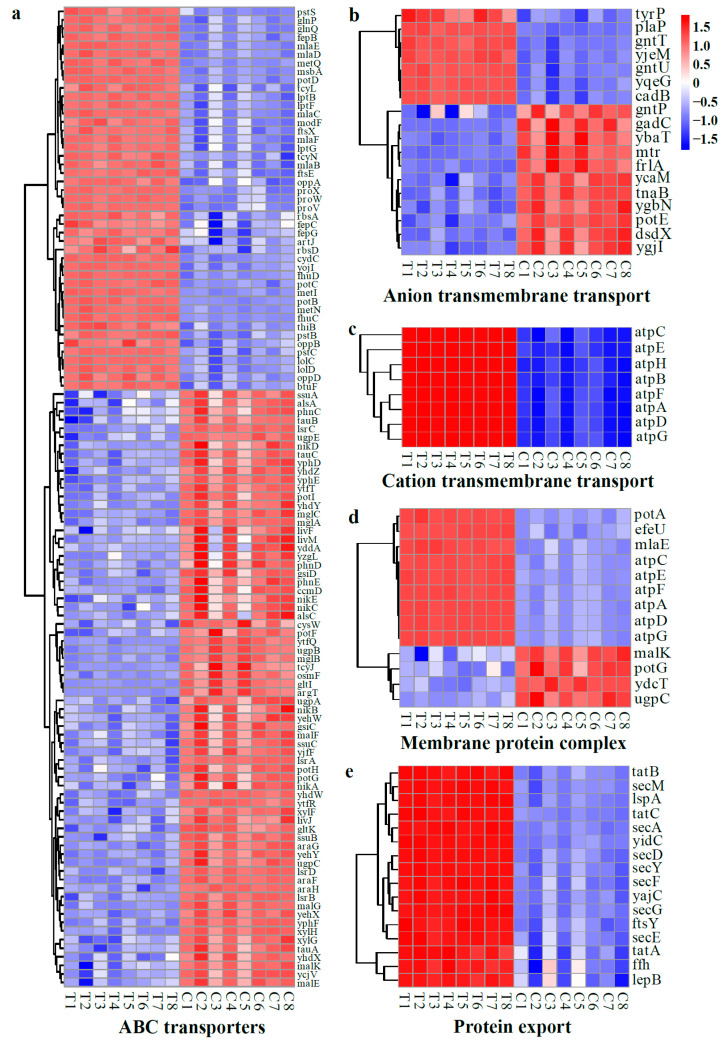
Heat map of typical DEGs related to cell membrane functions, including ABC transporters (**a**), anion transmembrane transport (**b**), cation transmembrane transport (**c**), membrane protein complex (**d**), and protein export (**e**). Red indicates up-regulated genes, while blue indicates down-regulated genes.

Changes in transporters, protein complexes, and protein export in the cellular membrane provide a clear picture of alterations in cellular membrane functionality. Following treatment with WGHa, several genes encoding amino acid transporters, such as L-arginine (*artJ*), L-glutamine (*glnP*), methionine (*metI*), cystine (*tcyL*/*N*), thiamine (*thiB*), glutathione/L-cysteine (*cydC*), glycine/betaine (*proV*/*W*/*X*), and tyrosine (*tyrP*) transporters, exhibited an up-regulation in expression (Figure 13a,b). Additionally, genes associated with ion transporters responsible for phosphate (*pstB*/*C*/*S*), iron (*fepB* and *fhuD*), and spermidine (*potB*/*C*/*D*) transport were also up-regulated. Moreover, genes encoding biomolecule transporters involved in LPS (*lptB*/*F* and *msbA*), phospholipid (*mlaB*/*C*/*D*/*E*/*F*), oligopeptide (*oppA*/*B*/*D*), and gluconate (*gntT*/*U*) transport demonstrated an increased expression level after WGHa treatment (Figure 13a,b). Conversely, genes encoding carbohydrate transporters of D-allose (*alsA*/*C*), arabinose (*araF*/*G*/*H*), maltose (*malE*/*F*/*G*/*K*), D-galactose (*mglA*/*B*/*C*), xylose (*xylF*/*G*/*H*), and galactofuranose (*yjfF* and *ytfQ*/*R*/*T*) were down-regulated (Figure 13a). Additionally, genes encoding ion transporters responsible for sulfate/thiosulfate (*cysW*), Ni^2+^ (*nikA*/*B*/*C*/*D*/*E*), and phosphonate (*phnC*/*D*/*E*, *ugpA*/*B*/*C*/*E*) transport were down-regulated (Figure 13a). Meanwhile, genes encoding membrane protein complexes, including ATP synthase (*atpA*/*B*/*C*/*D*/*E*/*F*/*G*/*H*) (Figure 13c,d), signal peptide recognition particle protein and signal peptidase (*ffh*, *ftsY*, *lepB*, and *lspA*), protein folding system (*tatA*/*B*/*C*), protein translocation subunit (*secA*/*D*/*E*/*F*/*G*/*M*/*Y* and *yajC*), and membrane protein insertase (*yidC*) complexes exhibited up-regulated expression levels, while putrescine (*potG*) was down-regulated (Figure 13e).

In *E. coli*, ATP-binding cassette (ABC) transporters primarily consist of import systems responsible for the ATP-driven translocation of ions, amino acids, nucleotides, polysaccharides, peptides, and even proteins. These transporters play an important role in regulating numerous physiological functions essential for maintaining cell homeostasis [48]. In this study, the WGHa treatment of *E. coli* resulted in the up-regulation of numerous genes related to amino acid and phospholipid transport, as well as protein translocation, processing, and export. Conversely, genes encoding carbohydrate transporters were down-regulated. The probable reason for this is that *E. coli* tries to maintain normal cellular physiological functions by enhancing protein translation under the stressful effects of WGHa. A previous study found that cell membrane damage triggers the initiation of cellular phospholipid self-repair [49]. However, the down-regulation of carbohydrate transporter genes leads to the insufficient synthesis of important functional molecules such as glycoproteins and LPS, ultimately leading to impaired cellular functions [50].

##### DEGs Related to Environmental Adaptation

**Figure 14:**
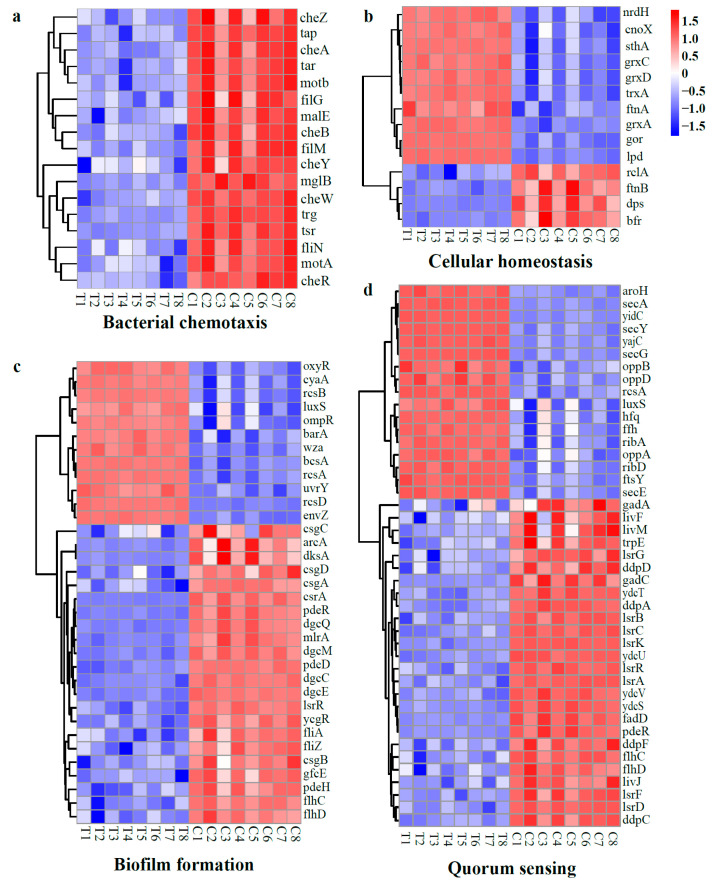
Heat map of typical DEGs related to environmental adaptation, including bacteria chemotaxis (**a**), cellular homeostasis (**b**), biofilm formation (**c**), and quorum sensing (**d**). Red indicates up-regulated genes, while blue indicates down-regulated genes.

In this study, the DGEs associated with bacterial chemotaxis were all down-regulated, including methyl-accepting chemotaxis proteins (*tap*, *tar*, *trg*, and *tsr*), chemotaxis proteins (*cheA*/*B*/*R*/*W*/*Y*/*Z*), flagellar motor switch proteins (*fliG*/*M*/*N*), and motility proteins (*motA*/*B*) (Figure 14a). Conversely, most DGEs associated with cellular homeostasis, including chaperedoxin (*cnoX*), glutathione reductase (*gor*), glutaredoxin (*grxA*/*C*/*D* and *nrdH*), and thioredoxin (*trxA*), were found to be up-regulated (Figure 14b). Bacterial chemotaxis refers to the directed movement of bacteria toward a favorable chemical gradient or away from a toxic one. This process relies on the sensing of a chemical gradient by chemoreceptors [51]. The down-regulation of DGEs related to chemotaxis indicates a compromised ability of the microbes to sense chemical cues and to navigate toward more favorable environments. Regarding cellular homeostasis, the up-regulation of genes encoding reductases suggests that WGHa induces oxidative stress in *E. coli*. Similar findings were reported in [43], where the authors observed an overexpression of genes related to the antioxidant defense system in *E. coli* following treatment with Au25 NCs.

The up-regulated DEGs associated with biofilm formation include those encoding sensory histidine kinase (*barA*, *envZ*), cellulose synthase (*bcsA*), adenylate cyclase (*cyaA*), and bacterial regulatory proteins (*csgD*, *ompR*/*oxyR*, *rcsA*, *rcsB*, and *uvrY*). In contrast, genes encoding diguanylate cyclase (*dgcC*/*E*/*M*/*Q*), curlin (*csgA*/*B*), flagellar brake protein (*ycgR*), flagellar transcriptional activator (*flhC*/*D*), and phosphodiesterases (*pdeD*/*H*/*R*) were down-regulated (Figure 14c). Biofilms are assemblages of microbial cells that form when bacteria become embedded within a matrix of self-produced protein, polysaccharide fibers, and extracellular DNA. This matrix offers protection to the microbes against unfavorable environmental conditions [52,53]. Several genes, including *bcsA*, *csgD*, *cyaA*, *csrA*, *fhlD*, and *fliA*, are known to play crucial roles in the early formation and development of biofilms in *E. coli* [54]. Notably, cellulose synthase encoded by *bcsA* is responsible for catalyzing the formation of cellulose, which serves as a physical and chemical barrier to protect the cell. The *csgD* gene regulates the production of curli fimbriae, positively influencing biofilm formation and stress regulation. The *cyaA* gene is indispensable for flagella synthesis, and mutations in *cyaA* resulted in severe biofilm defects and the loss of motility [55]. The *csrA* gene is involved in motility and flagellum biosynthesis, whereas *flhD* activates the class 3 flagellum operons, and *fliA* generates force to rotate the flagellar motor [54]. Based on these findings, we propose that WGHa interferes with biofilm formation while leading to a decline in cell motility and locomotion as a result of the down-regulation of genes related to flagellar synthesis.

DEGs pertinent to quorum sensing (QS) exhibited altered expression patterns in the present study (Figure 14d). Specifically, up-regulation was observed in S-ribosylhomocysteine lyase (*luxS*), Sec translocon (*secA*/*E*/*G*/*Y*), and oligopeptide transporters (*oppA*/*B*/*D*). Conversely, down-regulation was observed in genes encoding dipeptide transporters (*ddpA*/*B*/*C*/*D*/*F*), the RNA-binding protein Hfq (*hfq*), the autoinducer-2 transporter (*lsrA*/*B*/*C*/*D*/*K*), and a monooxygenase (*lsrG*) involved in antibiotic biosynthesis. Autoinducer-2 (AI-2) is a typical QS signaling molecule that mediates communication within and between many bacterial species. In *E. coli*, the *luxS* gene encodes S-ribosylhomocysteine lyase, an enzyme responsible for AI-2 synthesis. Additionally, specific transporters encoded by the *lsr* operons mediate the transmembrane transport of AI-2 [54,56]. In the present study, it was observed that, despite the up-regulation of the *luxS* gene and the potential for increased AI-2 synthesis, the down-regulation of transporter-encoding genes may lead to a decline in extracellular AI-2 levels. Consequently, this decrease has the capacity to impact normal QS function, disrupting the communication process among bacterial populations.

##### DEGs Related to Energy Metabolism

**Figure 15:**
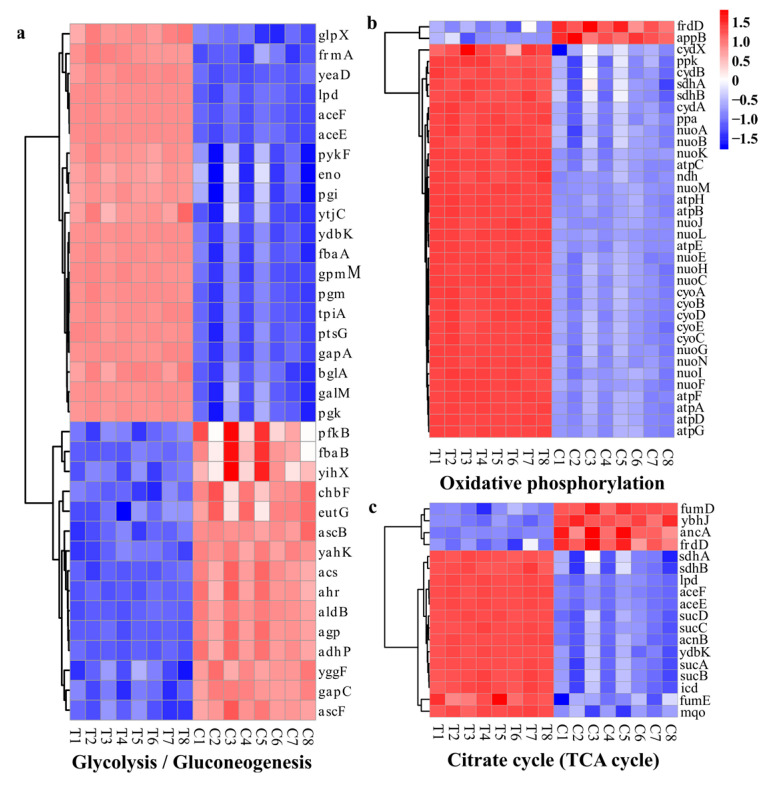
Heat map of typical DEGs related to energy metabolism, including those involved in glycolysis/gluconeogenesis (**a**), oxidative phosphorylation (**b**), and the citrate cycle (**c**). Red indicates up-regulated genes, while blue indicates down-regulated genes.

Environmental stresses can severely impact an organism’s energy balance, necessitating supplementary energy to restore or maintain equilibrium [43]. In the present investigation, most genes associated with the tricarboxylic acid cycle (TCA) and oxidative phosphorylation were up-regulated (Figure 15a,b). Several key enzyme genes such as the pyruvate kinase gene (*pykF*) in glycolysis (Figure 15a), the isocitrate dehydrogenase gene (*icd*), the 2-oxoglutarate dehydrogenase gene (*sucA*), and the dihydrolipoyltranssuccinylase gene (*sucB*) in the TCA were up-regulated (Figure 15c), whereas the fructose-1,6-bisphosphatase gene (*yggF*) involved in gluconeogenesis was down-regulated (Figure 15a). The membrane-embedded F_1_F_0_-ATP synthases are the key enzymes responsible for ATP synthesis. They employ the transmembrane proton gradients generated by the respiratory chain to synthesize ATP. There are eight genes (*atpA*/*B*/*C*/*D*/*E*/*F*/*G*/*H*) encoding different subunits of the F0 and F1 complexes of ATP synthase in bacteria [57]. The present study revealed a 20-fold up-regulation in the expression levels of these eight genes. However, despite the up-regulation, intracellular ATP levels significantly decreased following WGHa treatment, indicating that WGHa increased cell membrane permeability and intracellular ATP efflux and induced a compensatory up-regulation of ATP synthase genes to maintain cellular energy homeostasis. These findings are consistent with a previous report in which the authors observed that flavonoids from *Chimonanthus salicifolius* (FCS) can inhibit *S. aureus*, leading to increased expression levels of genes encoding the α and ε subunits of ATP synthase, thereby interfering with cellular energy metabolism [58]. Overall, the up-regulation of oxidative phosphorylation and TCA-associated genes demonstrates that *E. coli* combats environmental stress through heightened metabolism after WGHa treatment. Although this adaptive response partly mitigates the adverse effects of unfavorable environments, it also precipitates premature cellular aging and diminished metabolic viability [59].

##### DEGs Related to Nucleic Acid Synthesis and Repair

**Figure 16:**
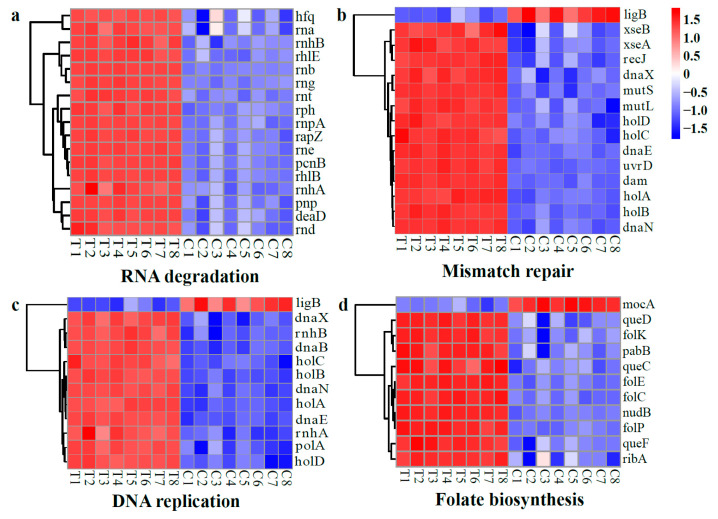
Heat map of typical DEGs related to nucleic acid synthesis and repair, including RNA degradation (**a**), mismatch repair (**b**), DNA replication (**c**), and folate biosynthesis (**d**). Red indicates up-regulated genes, while blue indicates down-regulated genes.

In this study, most of the DEGs associated with RNA degradation, DNA replication, mismatch repair, and folate biosynthesis were up-regulated, as illustrated in Figure 16. These up-regulated DEGs include genes that encode RNases (*rna*, *rnb*, *rnd*, *rne*, *rng*, *rnhA*/*B*, *rnpA*, *rnt*, and *rph*), an RNase adaptor protein (*rapZ*), and a PNPase (*pnp*) (Figure 16a). It is well known that RNase E (*rne*), RNase II (*rnb*), and PNPase (*pnp*) are the three key exonucleases involved in RNA degradation [60]. Additionally, the RNase adaptor protein (*rapZ*) was observed to activate RNase E through protein–protein interactions, thus facilitating the cleavage of small regulatory RNAs [61]. Meanwhile, the expression levels of genes encoding RNA helicase (*deaD* and *rhlB*/*E*), poly(A) polymerase I (*pcnB*), and RNA-binding protein (*hfq*) in the RNA degradation pathway were significantly up-regulated following WGHa treatment (Figure 16a). This suggests that WGHa disrupts RNA integrity by increasing the expression levels of enzymes involved in RNA degradation, which aligns with the previously observed decrease in RIN values of the RNA of WGHa-treated cells (Table S1).

DNA replication and repair systems are essential for ensuring the accurate duplication of the genome. In comparison to the control group, the WGHa treatment resulted in a significant up-regulation of numerous genes associated with these systems, including DNA helicase (*dnaB*), DNA polymerase (*dnaE*/*X* and *holA*/*B*/*C*/*D*), RNase H (*rnhA*/*B*), and DNA mismatch repair proteins (*mutL*/*S*) (Figure 16b,c). Therefore, this implies that *E. coli* enhances the expression levels of genes associated with DNA replication and repair systems in order to protect itself from adverse stress conditions. In agreement with our study, a previous report also observed the up-regulation of many genes involved in DNA replication and pyrimidine and purine metabolism upon FCS treatment [58]. Notably, excessive DNA replication will trigger replication stress, which has serious implications for DNA damage, genome stability, and cell survival [62].

Folate is a critical methyl donor in organisms, playing an important role not only in DNA synthesis but also in the maintenance of methylation reactions within cells [63]. In this study, the expression levels of several important enzymes related to folate synthesis such as dihydrofolate synthetase (*folC*), 2-amino-4-hydroxy-6-hydroxymethyldihydro- pteridine diphosphokinase (*folK*), dihydropteroate synthase (*folP*), and 6-pyruvoyl tetra- hydropterin synthase (*queD*) were up-regulated (Figure 16d). This finding suggests that WGHa may induce DNA damage and subsequently trigger accelerated folate synthesis in *E. coli*.

## 3. Materials and Methods

### 3.1. Strains and Antibiotics

The bacteria *S. aureus* CVCC2257, *B. subtilis* CVCC717, and *E. coli* CVCC1382 were obtained from the National Center for Veterinary Culture Collection (Wuhan, China). A 10% kitasamycin premix was purchased from Guangzhou Nongfeng Animal Pharmaceutical Co. (Guangzhou, China). Flavomycin (purity ≥ 99%) was purchased from North China Pharmaceutical Company Ltd. (Shijiazhuang, China). All other chemicals and solvents used in this study were of analytical grade and obtained from Sangon Biotech (Shanghai) Co., Ltd. (Shanghai, China).

### 3.2. Preparation of WGH Extracts

Mature, fresh walnut fruits were collected from Aksu, Xinjiang, China. The natural dried WGHs were crushed and passed through a 100-mesh sieve. The powders were then extracted using anhydrous ethanol, a 50% (*v*/*v*) hydroethanol solution, or deionized water. Extraction was carried out in a water bath shaker (ZWYR-200D, Shanghai Zhicheng Analytical Instrument Manufacturing Co., Shanghai, China) at a shaking speed of 170 rpm for 8 h, with a powder-to-solvent ratio of 1:10. The residue was removed, and the extracts were concentrated using a rotary evaporator (RE-52, Shanghai Yarong Biochemical Instrument Factory, Shanghai, China) and finally dried at 55 °C until a constant weight was achieved. The extracts obtained using anhydrous ethanol, 50% hydroethanol solution, and deionized water were labeled as WGHa, WGHb, and WGHc, respectively.

### 3.3. Antimicrobial Activity of WGH Extracts

#### 3.3.1. Diameter of Inhibition Zones

The antimicrobial effects of the WGH extracts against *S. aureus*, *B. subtilis*, and *E. coli* were evaluated using the Oxford cup method [64] with slight modifications. Briefly, a volume of 0.1 mL of bacterial culture (1 × 10^8^ colonies forming units (CFU)/mL) was added to 15 mL of sterilized Luria–Bertani (LB) agar medium and well mixed. An Oxford cup (Φ8.0 × 6 × 10 mm) was placed onto the solidified LB medium and was filled with 200 µL of a 200 mg/mL solution of the extracts. Positive controls were established using kitasamycin and flavomycin (both at 50 mg/mL), and negative controls used the respective extraction solvents. The dishes were incubated in an incubator (DHP-9162, Shanghai Yiheng Scientific Instrument Co., Shanghai, China) at 37 °C for 24 h. The diameter of the inhibition zones was measured. Triplicates were used in three independent assays (unless otherwise specified, the same as below).

#### 3.3.2. MIC

The MICs of the extracts were determined using the microdilution method with minor modifications [65]. Two-fold serial dilutions of the WGH extracts were added to sterilized LB agar medium, resulting in a final concentration of the extract ranging from 0.40 to 200 mg/mL. A volume of 0.2 mL of the extract-containing medium was pipetted into a sterile 96-well microplate, and 20.0 µL of the bacterial culture (1 × 10^8^ CFU/mL) was added to each well. The plates were then incubated at 37 °C for 24 h. The concentration at which the extract completely inhibited the growth of the bacteria was recorded as the MIC.

#### 3.3.3. Kinetics of Growth

The kinetic growth of the microorganisms in the presence of WGHa was evaluated as described in [66]. WGHa was added to 20.0 mL of sterilized LB medium to give a final concentration of 0, 0.25, 0.5, or 1.0 × MIC. A fresh bacterial culture (1 × 10^8^ CFU/mL) was inoculated at a final concentration of 0.1% in the growth medium. The mixture was then incubated at 37 °C and 170 rpm for 24 h. Every 2 h, a 0.2 mL volume of the culture was sampled, and the absorbance value at 600 nm (A600) was recorded using a spectrophotometer (UV-1800, Shimadzu, Kyoto, Japan).

### 3.4. Identification of Compounds in WGHa via Ultrahigh-Performance Liquid Chromatography–Tandem Mass Spectrometry (UHPLC-MS/MS)

Approximately 50 mg of WGHa was dissolved in 0.5 mL of a methanol/acetonitrile solution (1:1, *v*/*v*) and used for chromatographic analysis. UHPLC was performed on a UHPLC Ultimate 3000 (Thermo Fisher Scientific, Waltham, MA, USA) equipped with a Waters HSS T3 column (2.1 × 100 mm, 1.8 μm particle size, Waters, Wilmslow, UK) [67]. In positive ion mode, the mobile phase system consisted of water with 0.1% formic acid (buffer A) and acetonitrile with 0.1% formic acid (buffer B). In negative ion mode, the mobile phase system comprised water with 2 mM ammonium acetate (buffer A) and acetonitrile (buffer B). The gradient elution program was as follows: 0–2.5 min, 95% A; 2.5–14.0 min, 90% A; 14.0–22.0 min, 60% A; 22.0–23.1 min, 5%; 23.1–25 min, 95% A. The flow rate was 0.4 mL/min. The column temperature was maintained at 40 °C, and the injection volume for each sample was 5 μL.

Mass spectrometry (MS) was performed on a Triple TOF 5600+ (AB Sciex, Framingham, MA, USA). The data acquisition was performed separately in negative and positive electrospray ion (ESI) modes using Analyst TF 1.7 software (AB Sciex, USA). The MS survey scan range was from 50 to 1200 *m*/*z*, and the collision energy was set at 30 eV. The ESI ion source parameters were set as follows: nebulizer gas pressure, 60 psi; curtain gas pressure, 35 psi; and aux gas pressure, 60 psi. The heater temperature and capillary temperature were both set to 650 °C, and the ion spray voltage was set to 5.0 kV (positive ion mode) and −4.0 kV (negative ion mode).

The raw MS data were converted into the mzM format using ProteoWizard software (v 3.0) (Protein Metrics, Cupertino, CA, USA). Subsequently, the MS-DIAL software (v 4.9) was utilized for peak identification, peak filtration, and peak alignment. The data matrix including mass-to-charge ratio (*m*/*z*), retention time, and intensity was generated. Compound identification was first confirmed based on their accurate molecular weight, with a mass tolerance of no more than 30 ppm, and the collected MS/MS data were analyzed using the following metabolite databases: Massbank, GNPS, RIKEN PlaSMA, BMDMS-NP, mzClound, and self-developed databases.

### 3.5. Antimicrobial Mechanisms of WGHa

#### 3.5.1. Extracellular AKP Activity

The selected bacteria strains in the mid-log growth phase were inoculated into liquid LB medium to achieve a final concentration of 1 × 10^8^ CFU/mL (unless otherwise specified, this bacterial concentration was used for all subsequent experiments). WGHa was added to reach a final concentration of 1 × MIC (the same concentration was used for all subsequent experiments). The cultures were then incubated in a water bath shaker at 37 °C and 170 rpm for 8 h. At 0, 2, 4, 6, and 8 h, 1.0 mL of each culture was sampled and centrifuged at 4 °C and 12,000× *g* for 10 min. The supernatant was collected to determine the extracellular AKP activity using a kit according to the manufacturer’s instructions (Nanjing Jiancheng Bioengineering Institute Co., Ltd., Nanjing, China). To eliminate any interference caused by the WGHa extracts on the measurements, a blank medium containing the same amount of extract was used as a reference.

#### 3.5.2. Conductivity of the Medium, Intracellular ATP Content, and ATPase Activity

Liquid LB medium containing the selected bacteria and WGHa was incubated at 37 °C with shaking at 170 rpm. Samples (10.0 mL) were collected after 0, 2, 4, 6, 12, 18, and 24 h of incubation and then centrifuged. The supernatant was diluted twenty times with double-distilled water, and the conductivity of the diluted supernatant was measured using a conductivity meter (FE38, Mettler-Toledo, Shanghai, China). Furthermore, the conductivity of the diluted supernatant of the mixture of the LB medium and WGHa collected at the beginning of the incubation was also recorded and used as a negative control. Cell pellets were washed three times with sterile 0.1 M phosphate-buffered saline (PBS, pH 7.4) and resuspended in 10.0 mL PBS. The cells were disrupted using an ultrasonic cell crusher (JY92-IIN, Ningbo Scientz Biotechnology Co., Ltd., Ningbo, China) on ice (ultrasonic power of 200 W, ultrasonic time of 2 s, interval of 10 s, 40 cycles). After disruption and centrifugation, the resulting supernatant was collected to determine the contents of intracellular ATP and protein, and the activities of Na^+^/K^+^-ATPase, Mg^2+^-ATPase, and Ca^2+^-ATPase using a kit (Nanjing Jiancheng Bioengineering Institute Co., Ltd., China). A blank was also included.

#### 3.5.3. Loss of 260 nm Light-Absorbing Substances

Bacteria in the mid-log growth phase were centrifuged at 4 °C, 3000× *g* for 10 min. The resulting cell pellets were washed three times and resuspended in PBS. WGHa was added to the bacterial suspension, and the suspension was incubated at 37 °C and 170 rpm for 20 h. Subsequently, the bacterial suspension was centrifuged, and the resulting supernatant was carefully collected. To remove any impurities, the supernatant was filtered through a 0.45 µm membrane filter. The absorbance value of the filtered supernatant was measured at 260 nm, and a blank sample was used for comparison.

#### 3.5.4. Cellular Metabolic Vitality

Bacterial cultures were co-cultured with WGHa for 2 h. After that, the bacterial cultures were collected by centrifugation at 4 °C, 3000× *g* for 10. The resulting cell pellets were washed twice with sterilized saline and resuspended. INT was added to a final concentration of 1.0 mM, and the reaction was incubated at 37 °C for 30 min. The absorbance at 630 nm (A630) was subsequently measured.

#### 3.5.5. Fluorescence Intensity of Bacterial Nucleic Acids

Bacterial suspensions treated with WGHa were cultured at 37 °C and 170 rpm. A 0.8 mL volume of the bacterial culture was sampled at 0, 4, 8, 12, 18 and 24 h post-cultivation, and 2.4 mL of DAPI (Beyotime Biotech. Inc., Shanghai, China) was immediately added to achieve a final concentration of 5.0 μg/mL. The mixture was incubated at 23 °C and 170 rpm for 10 min. Subsequently, the fluorescence intensity of the bacterial DNA and RNA was determined using a fluorescence spectrophotometer (RF-5301pc, Shimadzu, Japan) with excitation wavelengths of 364 nm and 400 nm, and an emission wavelength of 454 nm.

#### 3.5.6. SEM

The bacteria suspensions treated with WGHa were incubated at 37 °C, 170 rpm for 20 h. Thereafter, 1.0 mL of the bacterial suspensions was sampled and centrifuged. A 1.0 mL volume of formaldehyde–acetic acid–alcohol fixative (FAA, consisting of formaldehyde 5%, acetic acid 5%, 50% alcohol 90%, *v*/*v*) was added to the cell pellets. The resuspended pellets were incubated at room temperature for 12 h to fix the cells. Afterward, the suspensions were centrifuged, and the resulting cell pellets were dehydrated using a series of ethanol concentrations (50, 60, 70, 75, 85, and 95%) for 15 min each. Subsequently, the dehydrated pellets were resuspended in 1.0 mL of tert-butanol and stored at 4 °C for 30 min until the tert-butanol solidified. The lyophilized cells were then sprayed and coated with platinum (Pt). The cell morphology was observed and photographed using a scanning electron microscope (Quanta FEG250, FEI, La Vergne, TN, USA) at an accelerating voltage of 2.00 kV and a magnification of 10,000×.

### 3.6. Transcriptome Sequencing of E. coli

WGHa was added to a suspension of *E. coli*, and the resulting culture was shaken at 37 °C and 170 rpm for 6 h. Subsequently, 3.0 mL of the culture was centrifuged, and the resulting cell pellets were then washed with sterile PBS. Total RNA was extracted using a commercial kit (DP 430, Tiangen Biotech (Beijing) Co., Ltd., Beijing, China) with eight biological replicates performed per treatment. The concentration of RNA was measured using a Nanodrop ND 1000 spectrophotometer (Thermo Fisher Scientific, MA, USA), and RNA integrity was assessed using an Agilent Bioanalyzer 2100 (Agilent Technologies, Palo Alto, CA, USA).

Poly(A)-containing mRNA was enriched using oligo (dT) beads and then fragmented using a mentation buffer. Complementary DNA (cDNA) was synthesized according to the method described in [68]. The purified double-stranded cDNA was end-repaired, A-tailed, and ligated to a sequencing adapter. The cDNA fragments of approximately 400 bp in length were purified using AMPure XP beads, and a sequencing library was subsequently constructed. The qualified libraries were sequenced on a Novaseq 6000 (Illumina, San Diego, CA, USA), and the paired-end reads with a length of 150 bp were generated. The Illumina sequences obtained from each sample were deposited into the short reads archive (SRA) with accession number PRJNA1010195.

The raw reads were quality filtered, and clean reads were obtained. The clean reads were aligned to the reference genome of *E. coli* ATCC 8739 (https://www.ncbi.nlm.nih.gov/datasets/genome/GCF_000019385.1/, accessed on 10 June 2023) using Bowtie 2.0 with default settings [69]. The number of reads that aligned to the predicted coding regions was calculated using HTSeq [70]. Gene expression levels were calculated and normalized to fragments per kilobases per million reads (FPKMs) using RSEM v1.3.1 (http://deweylab.github.io/RSEM/, accessed on 14 June 2023).

DEGs between control and WGHa-treated groups were identified using DESeq2 R v1.20.0 (http://bioconductor.org/packages/stats/bioc/DESeq2/, accessed on 15 June 2023) [71]. To control the false discovery rate, the *p*-value was adjusted using the Benjamini and Hochberg (BH) procedure. Genes with an adjusted *p* < 0.05 and |log_2_(fold change)| > 1 were considered differentially expressed. To investigate the functional implications of the DEGs, the GO and KEGG databases were utilized. The GOseq R package (v 1.54.0) [72] was employed for GO term enrichment analysis, while the KOBAS software (v 3.0) was used for the analysis of DEGs in KEGG pathways [73].

### 3.7. Statistics

Independent sample *t*-test or one-way ANOVA were performed using the IBM SPSS Statistics 19 (SPSS Inc., Chicago, IL, USA) software, and multiple comparisons between groups were performed using Duncan’s method. A *p*-value of less than 0.05 was considered statistically significant.

## 4. Conclusions

In this study, WGHa exhibited significant inhibitory activity against *S. aureus*, *B. subtilis*, and *E. coli*. The primary components identified were quercetin, syringic acid, and gallic acid. The antimicrobial mechanisms of WGHa included altering the cell membrane permeability and damaging the cell wall, resulting in the leakage of intracellular ions, proteins, and nucleic acids. This disruption ultimately led to the breakdown of cellular homeostasis. At the transcriptional level, WGHa diminished bacterial chemotaxis, motility, locomotion, and quorum-sensing capabilities. Additionally, it caused the up-regulation of TCA and oxidative phosphorylation genes, which interfered with cellular energy metabolism. Furthermore, WGHa compromised RNA integrity and induced DNA replication stress, consequently affecting the normal growth and proliferation of *E. coli*. This study provides a comprehensive understanding of the antimicrobial activity and mechanisms of WGH extracts and highlights the potential use of WGHa as a natural and effective antibacterial agent.

## Figures and Tables

**Table 1:** Inhibition zones (mm) of WGH extracts against *S. aureus*, *E. coli*, and *S. aureus*.

Bacteria	WGHa (200 mg/mL)	WGHb (200 mg/mL)	WGHc (200 mg/mL)	Kitasamycin (50 mg/mL)	Flavomycin (50 mg/mL)
*S. aureus*	18.08 ± 1.62 ^a^	12.50 ± 1.68 ^c^	0.00 ± 0.00 ^d^	11.92 ± 0.90 ^c^	16.50 ± 0.79 ^b^
*B. subtilis*	9.25 ± 1.36 ^c^	0.00 ± 0.00 ^d^	0.00 ± 0.00 ^d^	13.5 ± 1.00 ^a^	10.58 ± 0.99 ^b^
*E. coli*	3.75 ± 0.75 ^c^	0.00 ± 0.00 ^d^	0.00 ± 0.00 ^d^	7.08 ± 0.79 ^b^	11.00 ± 0.85 ^a^

Note: Values are expressed as mean ± standard deviation. The values with different superscript letters in the same row are significantly different (*p* < 0.05).

**Table 2:** Minimum inhibitory concentration (MIC) of WGH extracts against *S. aureus*, *E. coli*, and *S. aureus*.

Bacteria	MIC (mg/mL)			
	WGHa	WGHb	WGHc	Extraction Solvents
*S. aureus*	6.25	200	>200	>200
*B. subtilis*	6.25	>200	>200	>200
*E. coli*	25.00	>200	>200	>200

**Table 3:** The compounds in WGHa identified using the UHPLC-MS/MS method.

Compound Name	RT (min)	*m*/*z*	Adducts	Relative Content (%)	Classification	Formula
α-Cyperone	0.9220	241.1533	[M + Na]^+^	4.60	Terpenoid	C_15_H_22_O
Chlorogenic acid	2.5530	377.0841	[M + Na]^+^	2.64	Polyphenol	C_16_H_18_O_9_
Syringic acid	4.8165	199.0585	[M + H]^+^	7.60	Monophenol	C_9_H_10_O_5_
Gentiopicrin	5.2351	379.0995	[M + Na]^+^	1.29	Terpenoid	C_16_H_20_O_9_
Trans-ferulic acid	5.2351	177.0530	[M + H – H_2_O]^+^	2.90	Monophenol	C_10_H_10_O_4_
Myricetin	5.9873	319.0421	[M + H]^+^	1.61	Flavonoid	C_15_H_10_O_8_
Picroside II	6.6416	535.1387	[M + Na]^+^	1.36	Terpenoid	C_23_H_28_O_13_
Quercetin	6.9312	303.0491	[M + H]^+^	12.05	Flavonoid	C_15_H_10_O_7_
Taxifolin	7.2128	305.0643	[M + H]^+^	4.72	Flavonoid	C_15_H_12_O_7_
Cyanidin-3-glucoside	7.5995	449.1042	[M]^+^	2.17	Terpenoid	C_21_H_21_O_11_
Kaempferol-3-*O*-α-l-arabinoside	8.1943	419.0941	[M + H]^+^	3.82	Terpenoid	C_35_H_52_O_9_
Brucine	8.6146	395.2022	[M + H]^++^	1.86	Alkaloid	C_23_H_26_N_2_O_4_
Coniferyl aldehyde	8.7457	179.0691	[M + H]^+^	1.52	Monophenol	C_10_H_10_O_3_
6-Methylcoumarin	9.4412	161.0596	[M + H]^+^	3.48	Coumarin	C_10_H_8_O_2_
Licoricidin	10.3541	447.2166	[M + Na]^+^	2.24	Flavonoid	C_26_H_32_O_5_
Baccatin III	10.5884	609.2280	[M + Na]^+^	1.62	Terpenoid	C_31_H_38_O_11_
Dehydroandrographolide	17.1554	333.1991	[M + H]^+^	1.62	Terpenoid	C_20_H_28_O_4_
Fluoren-9-one	0.8525	179.0470	[M – H]^−^	8.95	Ketone	C_13_H_8_O
Succinic acid	1.2660	117.0127	[M – H]^−^	12.20	Dicarboxylic acid	C_4_H_6_O_4_
Gallic acid	1.4213	169.0070	[M – H]^−^	65.89	Polyphenols	C_7_H_6_O_5_

Note: RT, retention time; *m*/*z*, mass-to-charge ratio; relative content, the peak area of a compound/total peak area × 100%.

## Data Availability

The data presented in this study are available in the article and Supplementary Materials.

## Supplementary Materials

The following supporting information can be downloaded at https://www.mdpi.com/article/10.3390/molecules28247981/s1, Table S1. Effect of WGHe on concentration and quantity of total RNA of *E. coli.* Table S2. Data quality summary. Table S3. Results of samples mapped to the reference genome of *E. coli* ATCC 8739. Table S4. A complete description of the DEGs. Figure S1. (a) The total ion chromatograms in negative ion mode. (b) The total ion chromatograms in positive ion mode.
