# Supplementary material for: Antimicrobial Activity and Mechanisms of Walnut Green Husk Extract
Source: Molecules. 2023 Dec 7;28(24):7981. doi: 10.3390/molecules28247981 (PMC10745604; doi:10.3390/molecules28247981)
Supplement: Supplementary file 1 [file molecules-28-07981-s001.zip › Supplement Tables.docx]

**Table S1 Effect of WGHe on concentration and quantity of total RNA of *E. coli***

| Item | Treatment | Control |
| --- | --- | --- |
| Concentration (ng/μL) | 120.30±21.65^B^ | 158.20±26.11^A^ |
| rRNA ratio (23S/16S) | 1.19±0.06^b^ | 1.63±0.46^a^ |
| RIN | 5.85±0.08^B^ | 6.84±0.46^A^ |

Note: RIN: RNA integrity number.

**Table S2 Data quality summary**

| Sample | Raw reads | Clean reads | Clean bases | Error rate(%) | Q20 | Q30 | GC(%) |
| --- | --- | --- | --- | --- | --- | --- | --- |
| T1 | 13053558 | 12655328 | 1.90G | 0.02 | 98.56 | 95.53 | 52.57 |
| T2 | 15271856 | 14826938 | 2.23G | 0.02 | 98.50 | 95.38 | 52.58 |
| T3 | 12629758 | 12233102 | 1.84G | 0.02 | 98.56 | 95.51 | 52.56 |
| T4 | 14075776 | 13716204 | 2.06G | 0.02 | 97.99 | 94.19 | 52.61 |
| T5 | 15268638 | 14768970 | 2.22G | 0.02 | 98.53 | 95.46 | 52.56 |
| T6 | 15705312 | 15118570 | 2.27G | 0.02 | 98.02 | 94.16 | 52.62 |
| T7 | 15204910 | 14684568 | 2.21G | 0.02 | 98.56 | 95.52 | 52.60 |
| T8 | 14503252 | 13797684 | 2.07G | 0.02 | 98.50 | 95.41 | 52.65 |
| C1 | 13332258 | 13164028 | 1.98G | 0.02 | 98.54 | 95.44 | 52.68 |
| C2 | 13486236 | 13285266 | 2.00G | 0.02 | 98.58 | 95.53 | 52.39 |
| C3 | 13807362 | 13600814 | 2.05G | 0.02 | 98.63 | 95.64 | 52.67 |
| C4 | 15640076 | 15358974 | 2.31G | 0.02 | 98.41 | 95.04 | 52.54 |
| C5 | 16020654 | 15727128 | 2.36G | 0.02 | 98.51 | 95.35 | 52.55 |
| C6 | 15339498 | 15053712 | 2.26G | 0.02 | 98.63 | 95.63 | 52.43 |
| C7 | 15275322 | 14874990 | 2.24G | 0.02 | 98.45 | 95.20 | 52.28 |
| C8 | 13675090 | 13432944 | 2.02G | 0.02 | 98.66 | 95.72 | 52.28 |

Note: Sample: C1-C8: 0×MIC, T1-T8: 1×MIC; Raw reads: reads number in raw sequence data; Clean reads: valid reads after quality control; Clean bases: raw data filtered base number; Error rate (%): overall sequencing error rate of data; Q20: percentage of bases with Phred value > 20 in total bases; Q30:Phred value > percentage of bases with 30 in total bases; GC: percentage of G and C in four bases in clean reads.

**Table S3. Results of samples mapped to the reference genome of *E. coli* ATCC 8739**

| Sample | Clean reads | Total mapped (%) | Multiple mapped (%) | Uniquely mapped (%) |
| --- | --- | --- | --- | --- |
| T1 | 12655328 | 12370085 (97.75%) | 694741 (5.49%) | 11675344 (92.26%) |
| T2 | 14826938 | 14495083 (97.76%) | 816938 (5.51%) | 13678145 (92.25%) |
| T3 | 12233102 | 11955502 (97.73%) | 660995 (5.40%) | 11294507 (92.33%) |
| T4 | 13716204 | 13381911 (97.56%) | 776384 (5.66%) | 12605527 (91.90%) |
| T5 | 14768970 | 14427642 (97.69%) | 815416 (5.52%) | 13612226 (92.17%) |
| T6 | 15118570 | 14760158 (97.63%) | 864863 (5.72%) | 13895295 (91.91%) |
| T7 | 14684568 | 14361222 (97.8%) | 799482 (5.44%) | 13561740 (92.35%) |
| T8 | 13797684 | 13502212 (97.86%) | 773700 (5.61%) | 12728512 (92.25%) |
| C1 | 13164028 | 12869161 (97.76%) | 325635 (2.47%) | 12543526 (95.29%) |
| C2 | 13285266 | 12801273 (96.36%) | 461449 (3.47%) | 12339824 (92.88%) |
| C3 | 13600814 | 13316462 (97.91%) | 319402 (2.35%) | 12997060 (95.56%) |
| C4 | 15358974 | 14908703 (97.07%) | 458093 (2.98%) | 14450610 (94.09%) |
| C5 | 15727128 | 15370702 (97.73%) | 400248 (2.54%) | 14970454 (95.19%) |
| C6 | 15053712 | 14592952 (96.94%) | 450023 (2.99%) | 14142929 (93.95%) |
| C7 | 14874990 | 14463463 (97.23%) | 453529 (3.05%) | 14009934 (94.18%) |
| C8 | 13432944 | 13018983 (96.92%) | 392470 (2.92%) | 12626513 (94.00%) |

Note: Total mapped: the number of reads which can mapped to the reference genome; Multiple mapped: the number and percentage of reads with multiple alignment positions on the reference genome; Uniquely mapped: the number and percentage of reads with unique alignment positions on the reference genome.
