# Supplementary figures and images for: Antimicrobial Activity and Mechanisms of Walnut Green Husk Extract
Source: Molecules. 2023 Dec 7;28(24):7981. doi: 10.3390/molecules28247981 (PMC10745604; doi:10.3390/molecules28247981)

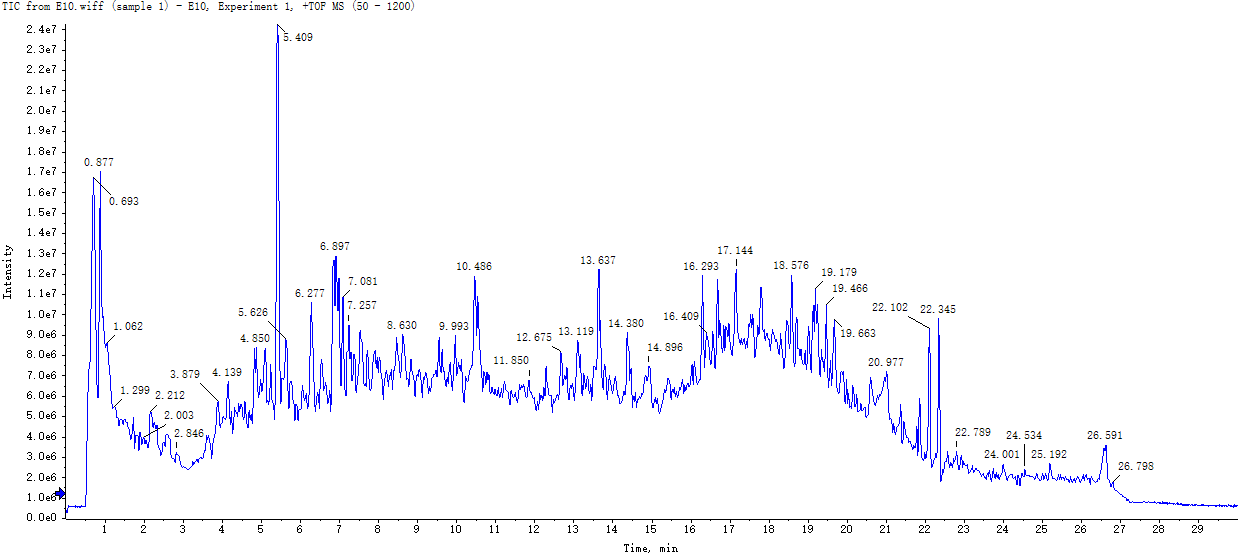

Supplement: Supplementary file 1 [file molecules-28-07981-s001.zip › Figure S1a The total ion chromatograms in negative ion mode.png]

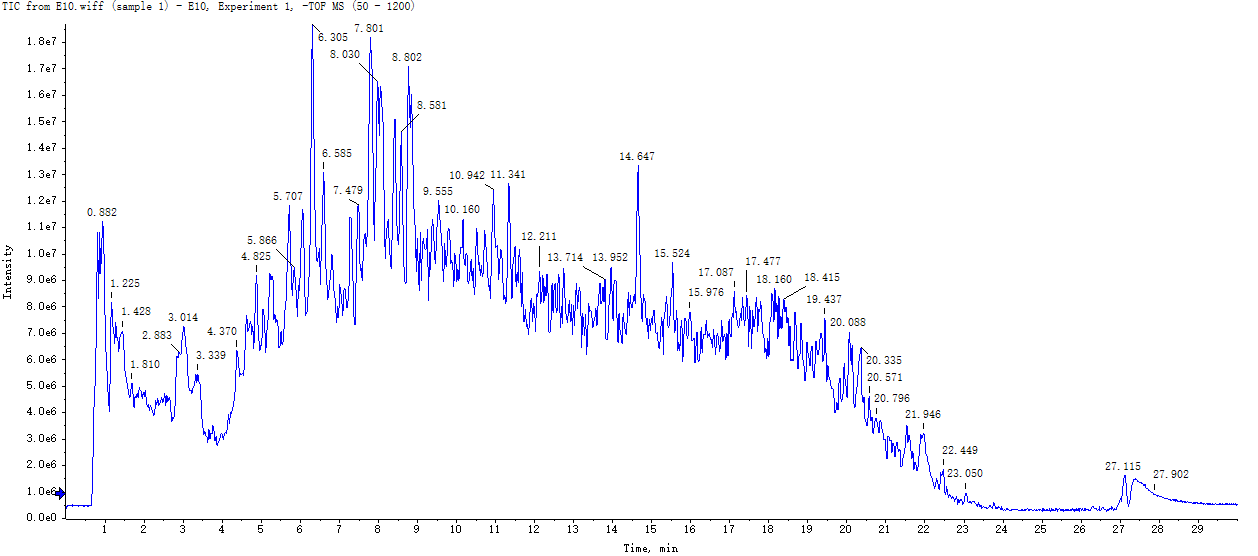

Supplement: Supplementary file 1 [file molecules-28-07981-s001.zip › Figure S1b The total ion chromatograms in positive ion mode.png]
